# Knowledge, attitudes, and practices of inpatients with chronic cardiovascular comorbidities regarding polypharmacy: a cross-sectional study

**DOI:** 10.3389/fphar.2025.1702721

**Published:** 2025-11-21

**Authors:** Lijun Xu, Chenggui Zhuo, Hongyan Xu, Zhelin Xia, Meicui Wu, Haipeng Cai

**Affiliations:** 1 Department of Pharmacy, Taizhou Central Hospital (Taizhou University Hospital), Taizhou, Zhejiang, China; 2 Department of Cardiology, Taizhou Central Hospital (Taizhou University Hospital), Taizhou, Zhejiang, China

**Keywords:** polypharmacy, chronic cardiovascular comorbidity, knowledge, attitudes, practice, medication adherence, drug-related side effects and adverse reactions, cross-sectional study

## Abstract

**Background:**

With the increasing prevalence of chronic cardiovascular diseases and the widespread use of multiple medications, polypharmacy has become a significant concern in patient management. This study aimed to examine the knowledge, attitudes, and practices (KAP) of patients with chronic cardiovascular comorbidities, such as coronary artery disease (CAD), hypertension, diabetes, and hyperlipidemia, concerning polypharmacy.

**Methods:**

A cross-sectional study was conducted among hospitalized patients from June 1 to 16 December 2024, utilizing a validated questionnaire-based survey to collect data. The self-made questionnaire included demographic information and an assessment of participants’ KAP scores related to polypharmacy.

**Results:**

A total of 706 valid responses were obtained. Of these, 493 participants (69.83%) were males, 342 (48.44%) were aged 65 years or older, and 347 (49.15%) resided in rural areas. The mean knowledge, attitude, and practice scores were 3.62 ± 2.52 (possible range: 0–7), 28.56 ± 3.97 (possible range: 10–50), and 17.55 ± 3.55 (possible range: 5–25), respectively. Structural equation modeling (SEM) further demonstrated that, among individuals taking five or more medications daily, knowledge exerted a positive direct effect on both attitude (β = 0.28, P < 0.001) and practice (β = 0.41, P < 0.001), while attitude also positively influenced practice (β = 0.39, P < 0.001). Moreover, an indirect pathway from knowledge to practice through attitude was suggested by the SEM results (β = 0.11, P < 0.001).

**Conclusion:**

Inpatients with chronic cardiovascular comorbidities exhibited insufficient knowledge and negative attitudes, with notable gaps in adherence behaviors regarding polypharmacy. These findings highlight the need for targeted educational interventions to enhance patients’ knowledge and attitudes toward polypharmacy, and to support safer and more consistent medication practices.

## Introduction

With global population aging and advances in medical care, chronic cardiovascular diseases have become increasingly prevalent worldwide, with cardiovascular comorbidities emerging as a leading cause of morbidity and mortality (Roth et al., 2020). Patients with conditions such as coronary artery disease (CAD), hypertension, diabetes, and hyperlipidemia often experience multiple concurrent cardiovascular risk factors, requiring complex medication regimens for optimal management (Bahiru et al., 2021). Studies indicate that up to 91% of patients in long-term care facilities take at least five medications daily, with cardiovascular patients representing a substantial proportion of this population (Jokanovic et al., 2015). This phenomenon of polypharmacy, defined as the regular use of five or more medications, has become particularly common among patients with chronic disease comorbidities. Diabetes, heart disease and other diseases are significantly associated with polypharmacy, and up to 64% of diabetic patients have polypharmacy, which is associated with hospitalization, emergency department visits and death (Chae et al., 2024; Hsu et al., 2021; Huang et al., 2021).

While polypharmacy is often clinically necessary for managing multiple chronic conditions, it presents significant challenges for both healthcare providers and patients (Halli-Tierney et al., 2019). To address the challenges posed by polypharmacy, it is essential not only to strengthen the review of chronic disease medications and optimize drug safety but also to assess patients’ capacity for self-managing their prescriptions. Polypharmacy is associated with increased risks of adverse drug events, decreased quality of life, increased mortality rates, and higher healthcare utilization (Hussain et al., 2025; Remelli et al., 2022).

The knowledge, attitude, and practice (KAP) model postulates that individual behaviors are contingent upon one’s knowledge and attitude. In the realm of public health, the examination of behavioral practices is commonly accompanied by the assessment of knowledge and risk perception, a practice frequently carried out through KAP surveys. This theory holds paramount significance in elucidating health-related behaviors (Vainauskienė and Vaitkienė, 2021). When applied to polypharmacy management, patients’ medication adherence and self-management behaviors might be influenced by their understanding of their medications and their attitudes toward complex treatment regimens. This relationship is especially relevant for patients with comorbid cardiovascular and cerebrovascular diseases, who are often prescribed multiple medications as part of long-term management plans. For these patients, insufficient knowledge can lead to misunderstandings about drug interactions or side effects, while negative attitudes may result in poor adherence, further increasing the risk of disease progression or complications.

Although many studies in China have explored polypharmacy, few have focused specifically on patients with chronic cardiovascular comorbidities-a population that accounts for a substantial proportion of morbidity and mortality among both urban and rural residents. Several regional studies in China have also reported high rates of polypharmacy and associated risks among patients with chronic diseases, underscoring the need for targeted investigations in this population (Liu et al., 2019; Shen and Zheng, 2024). Given that hypertension, diabetes, and hyperlipidemia are key risk factors for cardiovascular disease, our study targets patients with CAD or multiple CVD-related chronic conditions, aiming to investigate their knowledge, attitudes, and practices regarding polypharmacy. By distinguishing between patients experiencing polypharmacy and those who are not, this study explores crucial insight for developing tailored interventions that are beneficial for enhancing self-management of medication, reducing cardiovascular risk factors such as hypertension and diabetes, and ultimately contributing to improved long-term cardiovascular outcomes. Understanding these aspects is particularly crucial in the Chinese context, considering the unique healthcare system, cultural perceptions of medication, and the challenges posed by a rapidly aging population. However, little is known about the KAP of inpatients with chronic cardiovascular comorbidities regarding polypharmacy in China. Therefore, this study explicitly aimed to fill this gap by assessing their knowledge, attitudes, and practices through a cross-sectional survey.

## Materials and methods

### Study design and participants

This cross-sectional study was conducted at Taizhou Central Hospital (Taizhou University Hospital) from June 1 to 16 December 2024, involving patients with chronic disease comorbidities. We specifically recruited inpatients because their diagnoses and medication histories could be verified in detail through electronic medical records, which ensured higher accuracy of comorbidity and prescription data compared with outpatients. The inclusion criteria were as follows: adults aged 18–80 years with a confirmed diagnosis of at least two of the following conditions - hypertension, CAD, diabetes, and hyperlipidemia who had been using medications for chronic disease management for at least 3 months. Patients were excluded if they were critically ill, had mental disorders, had chronic diseases but did not receive pharmacological treatment. This study was approved by the Ethics Committee of Taizhou Central Hospital (Taizhou University Hospital) (approval number: 2024L-04-03), and all participants provided written informed consent. Initially, both inpatients and outpatients were considered for recruitment. However, in tertiary hospitals, the number of outpatients is extremely large, and completing a detailed questionnaire during clinical visits is difficult due to limited consultation time and multiple examinations. Moreover, most patients with chronic cardiovascular diseases are older and often followed up by telephone or online, resulting in very low response rates and reduced data accuracy. Therefore, we focused on inpatients, as researchers could provide real-time clarification of questionnaire items, thereby ensuring data completeness and accuracy.

### Questionnaire introduction

The final questionnaire, developed in Chinese (the English version can be found in the Appendix file), consists of four sections: demographic information, knowledge, attitude, and practice dimensions. The questionnaire was developed with reference to the Chinese Expert Consensus on the Evaluation and Management of Polypharmacy in the Elderly (Shen and Zheng, 2024), and was revised based on the feedback from six senior experts. These included two chief physicians in cardiology, one chief physician in endocrinology, one deputy chief physician in geriatrics, and two senior clinical pharmacists. The knowledge dimension includes seven questions, each scored as 1 point for a correct response and 0 points for an incorrect or “unsure” response, resulting in a total possible score ranging from 0 to 7. The attitude dimension comprises ten items assessed on a five-point Likert scale, with responses ranging from “strongly disagree” (1 point) to “strongly agree” (5 points), yielding a total score range of 10–50. The practice dimension consists of five items, also evaluated using a five-point Likert scale, where responses range from “never” (1 point) to “always” (5 points), leading to a total score range of 5–25. Adequate knowledge, positive attitudes, and proactive practices were defined as scores exceeding 70% of the maximum possible score in each respective section (Salman et al., 2020). During questionnaire administration, all medications currently used by participants were recorded, and topical drugs were excluded at the time of data collection and analysis to ensure that only systemic medications prescribed for chronic disease management were considered. In this study, proactive practices referred to higher practice scores (≥70% of the maximum), which indicate active engagement in recommended medication behaviors such as following prescriptions, avoiding missed doses, and checking medication instructions.

### Questionnaire distribution and validation

Eligible patients with cardiovascular chronic disease comorbidities were recruited from inpatients in the cardiology and endocrinology departments. Prior to enrollment, clinical pharmacists reviewed each patient’s diagnosis and medication history through electronic medical records. In cases where documentation was ambiguous or incomplete, a multidisciplinary team-including cardiologists, endocrinologists, and geriatricians-collaboratively verified the diagnosis to ensure accuracy. During hospitalization, patients’ blood pressure, glucose, and lipid levels were routinely monitored by attending physicians to assess treatment effectiveness. Trained researchers administered the questionnaires primarily through face-to-face interviews to ensure patient comprehension and accuracy in responses. Before data collection, patients were informed about the study and invited to participate voluntarily. Those who were unable to communicate, refused to participate, declined to answer questions, or were deemed clinically unsuitable were excluded.

A pilot test was conducted with 43 patients to evaluate the feasibility and reliability of the questionnaire, and all responses were considered valid. The pilot results demonstrated acceptable internal consistency, with an overall Cronbach’s α coefficient of 0.7486, and subscale reliability coefficients of 0.7000 (knowledge), 0.7107 (attitude), and 0.6458 (practice). Following refinement, the final survey was formally administered. A total of 731 questionnaires were distributed, and after excluding 25 due to incomplete or inconsistent responses, 706 complete and valid questionnaires were included in the final analysis. Excluded cases were removed solely due to questionnaire incompleteness, without systematic differences in demographic or clinical characteristics compared with included participants. The revised instrument showed improved internal consistency, with an overall Cronbach’s α of 0.8753 and subscale coefficients of 0.7742 (knowledge), 0.8333 (attitude), and 0.7624 (practice). Additionally, the Kaiser-Meyer-Olkin (KMO) value was 0.905 (P < 0.001), indicating high sampling adequacy for factor analysis. These results confirm that the instrument demonstrated acceptable reliability and construct validity, consistent with recommended methodological standards for scale development and psychometric testing (Morgado et al., 2017; Boateng et al., 2018).

### Sample size calculation

Sample size was calculated using the formula for cross-sectional studies (Naing et al., 2006): α = 0.05, $n={\left(\frac{Z_{1-\alpha /2}}{\delta }\right)}^{2}\times p\times \left(1-p\right)$ where $Z_{1-\alpha /2}$ = 1.96 when α = 0.05, the assumed degree of variability of p = 0.5 maximizes the required sample size, and δ is admissible error (which was 5% here). The theoretical sample size was 480 which includes an extra 20% to account for potential non-response or incomplete questionnaires.

### Statistical analysis

Statistical analyses were performed using STATA 17.0 (StataCorp, College Station, TX, United States). Continuous variables were presented as means and standard deviations (SD) and tested for normality. Normally distributed data were compared using one-way analysis of variance (ANOVA), while non-normally distributed data were analyzed using non-parametric tests, including the Kruskal–Wallis test for multiple group comparisons. Categorical variables were expressed as frequencies and percentages (n, %). Spearman’s rank correlation analysis was used to assess relationships among knowledge, attitude, and practice scores. Multivariate analysis was conducted using stepwise forward linear regression, with statistical significance set at P < 0.05. Structural equation modeling (SEM) was employed to explore interrelationships among KAP scores, with model fit assessed using the root mean square error of approximation (RMSEA), incremental fit index (IFI), Tucker–Lewis index (TLI), and comparative fit index (CFI). Model fit was judged according to commonly accepted threshold values: RMSEA <0.08, IFI >0.90, TLI >0.90, and CFI >0.90, which indicate an acceptable fit (Goretzko et al., 2024). The mediating effect of attitude between knowledge and practice was examined using the bootstrapping method with 1,000 resamples in AMOS. The indirect effect was considered significant if the 95% bootstrap confidence interval (CI) did not include zero. A two-tailed P-value <0.05 was considered statistically significant.

## Results

### Basic information of the population

Of the 706 participants, the majority were male (69.83%), aged 65 years or older (48.44%), and had primary school education or below (57.93%). Most had hypertension (84.28%) and diabetes (62.32%), with 71.53% taking five or more medications daily (Table 1).

**TABLE 1:** Basic characteristics and knowledge, attitude and practice dimensions.

Variables	N (%)	Knowledge		Attitude		Practice	
		Mean ± SD	P	Mean ± SD	P	Mean ± SD	P
Total score	***N* = 706**	3.62 ± 2.52		28.56 ± 3.97		17.55 ± 3.55	
Gender			**<0.001**		0.141		**0.011**
Male	493 (69.83)	3.92 ± 2.50		28.72 ± 3.88		17.76 ± 3.62	
Female	213 (30.17)	2.89 ± 2.40		28.19 ± 4.13		17.04 ± 3.31	
Age (years old)[range:24–80]			**<0.001**		**<0.001**		0.276
<60	225 (31.87)	4.56 ± 2.29		27.71 ± 3.85		17.29 ± 3.73	
60–64	139 (19.69)	4.21 ± 2.17		28.55 ± 4.03		17.94 ± 3.51	
≥65	342 (48.44)	2.74 ± 2.50		29.12 ± 3.93		17.54 ± 3.42	
Residence			**<0.001**		0.402		0.206
Rural	347 (49.15)	2.82 ± 2.30		28.38 ± 4.14		17.37 ± 3.47	
Urban	250 (35.41)	4.79 ± 2.43		28.76 ± 3.93		17.91 ± 3.58	
Suburban	109 (15.44)	3.41 ± 2.37		28.65 ± 3.47		17.23 ± 3.63	
Education			**<0.001**		0.695		**0.001**
Primary school or below	409 (57.93)	3.41 ± 2.37		28.65 ± 3.47		17.23 ± 3.63	
Middle school	174 (24.65)	2.59 ± 2.11		28.52 ± 3.96		17.10 ± 3.40	
High school/technical school or above	123 (17.42)	4.52 ± 2.30		28.53 ± 4.17		18.02 ± 3.42	
Occupation			**<0.001**		0.187		**0.009**
Government/Enterprise Administrator	43 (6.09)	5.72 ± 2.25		28.71 ± 3.68		18.34 ± 3.96	
Professional (e.g., teacher, doctor, engineer, writer, etc.)	59 (8.36)	6.16 ± 1.54		29.23 ± 3.40		19.25 ± 3.49	
General staff	69 (9.77)	6.33 ± 2.50		29.13 ± 4.17		18.49 ± 4.38	
Business/Service Industry Personnel	152 (21.53)	4.82 ± 2.24		28.57 ± 4.10		17.84 ± 3.38	
Production, Transportation, Equipment Operators	66 (9.35)	3.80 ± 2.08		28.63 ± 4.05		17.26 ± 3.53	
Agricultural, Forestry, Animal Husbandry, Fishery, and Water Conservancy Workers	230 (32.58)	3.98 ± 2.23		27.42 ± 3.47		17.65 ± 3.57	
Unemployed or Homemaker	87 (12.32)	2.36 ± 1.89		28.53 ± 4.02		17.12 ± 3.29	
Monthly income *per capita*			**<0.001**		0.218		**0.001**
<2000	139 (19.69)	1.82 ± 2.19		28.79 ± 3.95		17.22 ± 3.42	
2000–5,000	210 (29.75)	2.65 ± 1.97		28.14 ± 3.97		16.87 ± 3.28	
5,000–10000	243 (34.42)	4.50 ± 2.12		28.60 ± 4.05		17.93 ± 3.49	
>10,000	114 (16.15)	5.67 ± 2.29		28.96 ± 3.77		18.34 ± 3.98	
Marital status			0.067		0.904		0.179
Single	53 (7.51)	3.05 ± 2.67		28.56 ± 4.05		16.71 ± 4.02	
Married	653 (92.49)	3.66 ± 2.50		28.56 ± 3.96		17.61 ± 3.49	
Disease (multiple choice,at least 2)							
Hypertension	595 (84.28)						
Diabetes	440 (62.32)						
Hyperlipidemia	333 (47.17)						
Coronary heart disease	403 (57.08)						
Family member with above disease			0.816		**0.029**		**0.001**
Yes	316 (44.76)	3.61 ± 2.59		28.19 ± 3.72		17.06 ± 3.57	
No	390 (55.24)	3.62 ± 2.46		28.85 ± 4.13		17.93 ± 3.47	
Taking five or more medications daily			**0.006**		0.487		**<0.001**
Yes	505 (71.53)	3.75 ± 2.48		28.48 ± 3.79		17.91 ± 3.29	
No	201 (28.47)	3.26 ± 2.57		28.74 ± 4.38		16.60 ± 3.96	
Duration of chronic disease medication			0.530		0.270		**<0.001**
≤1 year	74 (10.48)	3.71 ± 2.59		27.74 ± 3.99		19.01 ± 3.77	
2 years–5 years	212 (30.03)	3.39 ± 2.31		28.54 ± 3.96		17.26 ± 3.49	
6 years–9 years	137 (19.41)	3.70 ± 2.35		28.52 ± 4.17		17.59 ± 3.57	
≥10 years	283 (40.08)	3.71 ± 2.71		28.80 ± 3.85		17.34 ± 3.42	
Medication taking (multiple choice)							
Antihypertensive drugs	572 (81.02)						
Antidiabetic drugs	410 (58.07)						
Lipid-lowering drugs	499 (70.68)						
Antiplatelet drugs (e.g., aspirin)	414 (58.64)						
Antianginal drugs	224 (31.73)						
Other drugs	326 (46.18)						
Adverse reaction in the past 6 months			0.497		0.103		0.333
Yes	95 (13.46)	3.44 ± 2.40		28.08 ± 3.90		17.65 ± 3.11	
No	605 (85.69)	3.63 ± 2.54		28.60 ± 3.96		17.50 ± 3.61	
Uncertain	6 (0.85)	4.66 ± 2.25		31.33 ± 4.67		19.5 ± 2.42	
Type of health insurance			**<0.001**		0.087		**<0.001**
New rural cooperative medical scheme	336 (47.59)	2.27 ± 1.89		28.27 ± 4.12		16.97 ± 3.35	
Employee medical insurance	137 (19.41)	6.09 ± 2.14		28.43 ± 3.77		18.54 ± 3.75	
Urban resident medical insurance	228 (32.29)	4.14 ± 2.22		29.08 ± 3.83		17.81 ± 3.54	
No insurance	5 (0.71)	2 ± 1		28 ± 3.08		16.4 ± 3.50	
Attending physician or family doctor			**<0.001**		**<0.001**		**<0.001**
Yes	133 (18.84)	4.89 ± 2.73		31.14 ± 4.00		20.03 ± 2.48	
No	573 (81.16)	3.31 ± 2.37		27.96 ± 3.71		16.96 ± 3.50	

### Distribution of knowledge, attitude, and practice responses

The distribution of knowledge dimensions showed that the three questions with the highest number of participants choosing the “Unaware” option were “Do you know that interactions can occur between different medications?” (K4) with 84.42%, “Polypharmacy generally refers to the routine use of five or more medications simultaneously.” (K1) with 83.43%, and “Do you know how to handle situations such as missed doses, incorrect doses, or repeated doses?” (K5) with 65.58%. Responses to the attitude dimension showed that 19.41% strongly agreed and 44.9% agreed that they worry that taking so many medications will harm their liver and kidney function (A8), 13.31% strongly agreed and 35.27% agreed that reducing the number of medications will better manage their health (A6), and 6.37% strongly agreed and 53.82% agreed that their life cannot function without these medications (A4). Responses to the practice dimension showed that 23.23% rarely and 50.99% never read the medication instructions to understand side effects and precautions (P5), 16.01% rarely and 2.83% never follow the timing and frequency of medication as instructed by my doctor (P1). Meanwhile, 11.33% often forget to take their medication (P2) (Supplementary Table S1).

### Correlation analysis of knowledge, attitude, and practice

Knowledge scores varied significantly across demographic and socioeconomic factors. Attitude scores varied significantly by age and healthcare access. Practice scores were associated with multiple demographic and socioeconomic factors (Table 1).

For total population, correlation analysis indicated significant positive correlations between knowledge and attitude (r = 0.1515, P < 0.001), as well as practice (r = 0.3628, P < 0.001). Meanwhile, there was also correlation between attitude and practice (r = 0.5583, P < 0.001). For those taking five or more medications daily, the correlation between KAP was similar to that of the total population. However, for those taking less than five medications daily, the correlation between knowledge and attitude was not significant (r = 0.1145, P = 0.1056) (Table 2).

**TABLE 2:** Correlation analysis.

	Knowledge	Attitude	Practice
Total participants			
Knowledge	1		
Attitude	0.1515 (P < 0.001)	1	
Practice	0.3628 (P < 0.001)	0.5583 (P < 0.001)	1
Taking five or more medications daily			
Knowledge	1		
Attitude	0.1676 (P < 0.001)	1	
Practice	0.3760 (P < 0.001)	0.5113 (P < 0.001)	1
Taking less than five medications daily			
Knowledge	1		
Attitude	0.1145 (P = 0.1056)	1	
Practice	0.3032 (P < 0.001)	0.6784 (P < 0.001)	1

### Multivariate linear regression for practice dimensions

Multivariate linear regression showed that knowledge score (Coef. = 0.37, 95% CI: [0.293–0.45], P < 0.001), attitude score (Coef. = 0.43, 95% CI: [0.374–0.47], P < 0.001), being married (Coef. = 0.88, 95% CI: [0.139–1.61], P = 0.02), and having no family member with the specified disease (Coef. = 0.59, 95% CI: [0.184–0.98], P = 0.004) showed positive associations with practices, while not taking five or more medications daily (Coef. = −1.28, 95% CI: [-1.71 to −0.84], P < 0.001), duration of chronic disease medication use for 2–5 years (Coef. = −1.77, 95% CI: [-2.46 to −1.06], P < 0.001), 6–9 years (Coef. = −1.74, 95% CI: [-2.48 to −0.99], P < 0.001), or ≥10 years (Coef. = −2.02, 95% CI: [-2.70 to −1.34], P < 0.001), and the absence of an attending physician or family doctor (Coef. = −1.02, 95% CI: [-1.55 to −0.48], P < 0.001) were negatively associated with practices (Table 3).

**TABLE 3:** Multivariate linear regression analysis for practice dimension.

Variables	Coef	95% CI	P
Adj R-squared = 0.4580, F = 67.19(P < 0.001)			
Knowledge score	0.37	0.293–0.45	<0.001
Attitude score	0.43	0.374–0.47	<0.001
Gender			
Male			
Female	-	-	-
Age (years old)[range:24∼81]			
<60			
60–64	-	-	-
≥65	-	-	-
Residence			
Rural			
Urban	-	-	-
Suburban	-	-	-
Education			
Primary school or below			
Middle school	-	-	-
High school/technical school or above	-	-	-
Occupation			
Government/Enterprise Administrator			
Professional (e.g., teacher, doctor, engineer, writer, etc.)	-	-	-
General staff	-	-	-
Business/Service Industry Personnel	-	-	-
Production, Transportation, Equipment Operators	-	-	-
Agricultural, Forestry, Animal Husbandry, Fishery, and Water Conservancy Workers	-	-	-
Unemployed or Homemaker	-	-	-
Monthly income *per capita*			
<2000			
2000–5,000	-	-	-
5,000–10000	-	-	-
>10,000	-	-	-
Marital status			
Single			
Married	0.88	0.139–1.61	0.02
Family member with above disease			
Yes			
No	0.59	0.184–0.98	0.004
Taking five or more medications daily			
Yes			
No	−1.28	−1.71–−0.84	0
Duration of chronic disease medication			
≤1 year			
2 years–5 years	−1.77	−2.46–−1.06	0
6 years–9 years	−1.74	−2.48–−0.99	0
≥10 years	−2.02	−2.70–−1.34	0
Adverse reaction in the past 6 months			
Yes			
No	-	-	-
Uncertain	-	-	-
Type of health insurance			
New rural cooperative medical scheme			
Employee medical insurance	-	-	-
Urban resident medical insurance	-	-	-
No insurance	-	-	-
Attending physician or family doctor			
Yes			
No	−1.02	−1.55–−0.48	0

### Structural equation model

**FIGURE 1:**
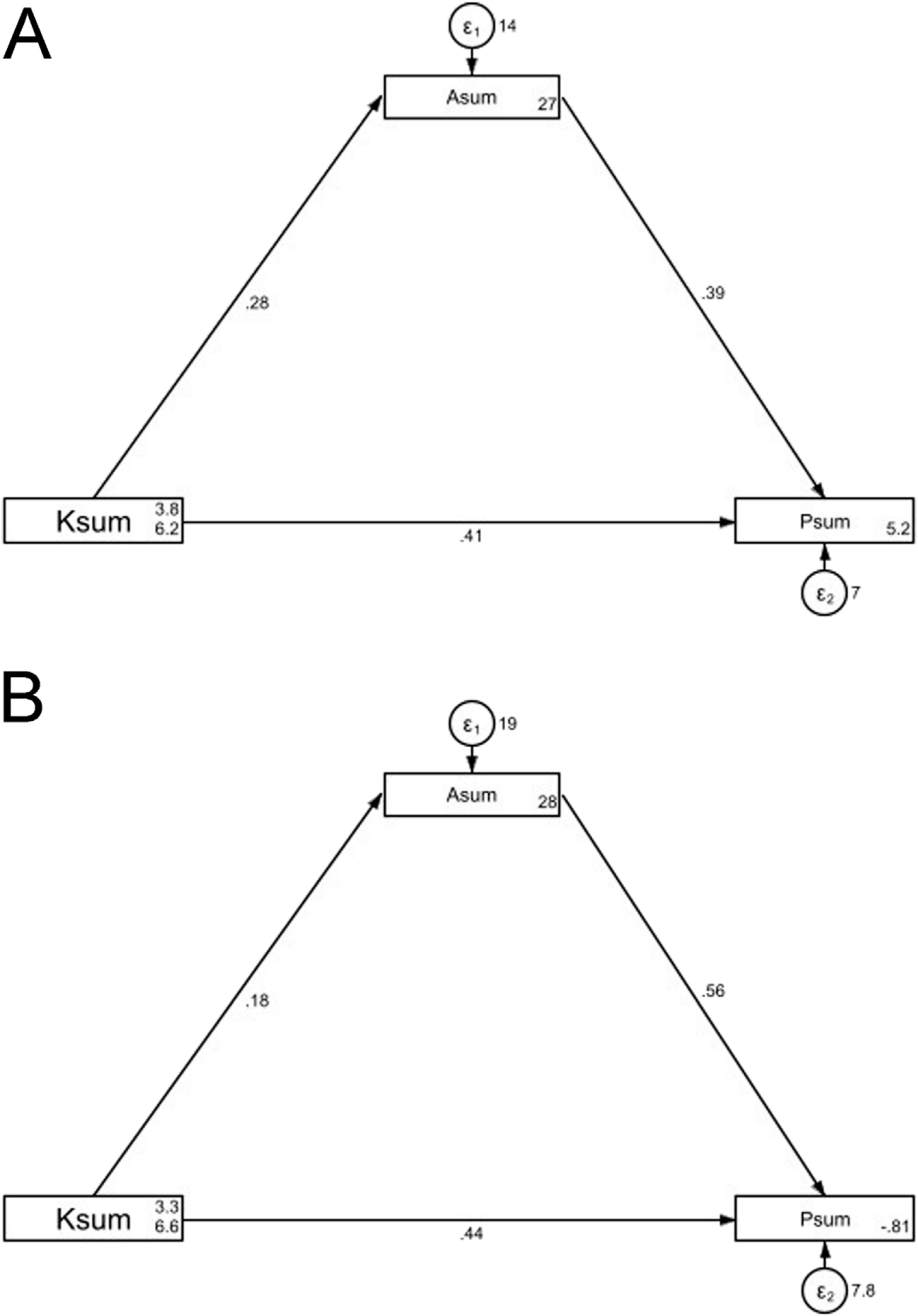
SEM model. **(A)** “Individuals taking five or more medications daily”. **(B)** “Individuals taking less than five medications daily”.

For those taking five or more medications daily, the SEM demonstrate a highly favorable model fit indices (RMSEA value: 0.000, SRMR value: 0.000, TLI value: 1.000, and CFI value: 1.000), suggesting a well-fitting model (Supplementary Table S2). In the SEM coding, Ksum, Asum, and Psum denote the composite scores for knowledge, attitude, and practice, respectively. SEM results show that knowledge had a positive direct effect on attitude (β = 0.28, P < 0.001) and a positive direct effect on practice (β = 0.41, P < 0.001). Meanwhile, attitude had a positive direct impact on practice (β = 0.39, P < 0.001). Furthermore, knowledge indirectly and positively affected practice through attitude (β = 0.11, P < 0.001) (Table 4; Figure 1A). However, comparing to the SEM results above, the direct effect of knowledge on attitude (β = 0.18, P = 0.132) and the indirect effect on practice were not significant anymore (β = 0.10, P = 0.135) (Table 4; Figure 1B).

**TABLE 4:** Mediation analysis.

Model paths		Total effects		Direct effect		Indirect effect	
		β (95% CI)	P	β (95% CI)	P	β (95% CI)	P
Individuals taking five or more medications daily							
Asum < -							
	Ksum	0.28 (0.15,0.41)	<0.001	0.28 (0.15,0.41)	<0.001	--	--
Psum < -							
	Asum	0.39 (0.32,0.45)	<0.001	0.39 (0.32,0.45)	<0.001	--	--
	Ksum	0.52 (0.41,0.62)	<0.001	0.41 (0.31,0.50)	<0.001	0.11 (0.05,0.16)	<0.001
Individuals taking less than five medications daily							
Model paths		Total effects	Direct effect	Indirect effect			
		β (95% CI)	P	β (95% CI)	P	β (95% CI)	P
Asum < -							
	Ksum	0.18 (-0.05,0.41)	0.132	0.18 (-0.05,0.41)	0.132	--	--
Psum < -							
	Asum	0.55 (0.46,0.64)	<0.001	0.55 (0.46,0.64)	<0.001	--	--
	Ksum	0.54 (0.34,0.74)	<0.001	0.44 (0.29,0.59)	<0.001	0.10 (-0.03,0.23)	0.135

Ksum, Asum, and Psum denote the composite scores for knowledge, attitude, and practice, respectively.

## Discussion

Patients with chronic cardiovascular diseases such as CAD and hypertension often require lifelong treatment with multiple medications, creating challenges in adherence and safety. This study found that these patients demonstrated inadequate knowledge, negative attitudes, yet relatively proactive practices concerning polypharmacy. The associations among knowledge, attitude, and practice varied according to the number of medications taken daily, with stronger effects observed in patients taking five or more drugs. These findings highlight the importance of targeted educational strategies for patients with complex regimens (Roth et al., 2020; Halli-Tierney et al., 2019). This is consistent with recent Chinese evidence showing that polypharmacy is common and often insufficiently managed among older patients with cardiovascular diseases (Shen and Zheng, 2024; Tomida et al., 2024). Although the correlations among KAP scores were statistically significant, some coefficients (e.g., r = 0.15) indicated only weak associations. These findings should be interpreted with caution, as statistical significance may largely reflect the relatively large sample size rather than a strong practical relationship.

Three main observations emerged. First, SEM findings suggested that knowledge may influence practice via attitude among those with polypharmacy, suggesting that patients on complex regimens are more responsive to education (Clark et al., 2020; Kamau et al., 2024). Second, patients taking fewer medications reported poorer adherence. A possible explanation is that patients prescribed fewer medications may perceive their disease risk as lower and therefore pay less attention to strict adherence, whereas patients on more complex regimens may be more aware of the risks and more likely to follow medical advice closely. This interpretation is supported by previous evidence showing that higher perceived risk and regimen complexity are associated with better adherence (Cross et al., 2020; Chantzaras and Yfantopoulos, 2023). This interpretation is consistent with previous evidence (Cross et al., 2020). In addition, several knowledge items were phrased as “Do you know … ?”, which may measure self-perceived awareness rather than objective knowledge. This design could have led to an overestimation of actual knowledge levels due to guessing or social desirability bias. Third, family support and professional guidance strongly influenced practice: being married, having family members with chronic diseases, and receiving advice from family doctors were all associated with better adherence (Chantzaras and Yfantopoulos, 2023; Jarrah et al., 2023).

Regression analysis confirmed that knowledge and attitudes were positively associated with practices, while longer treatment duration and lack of physician support were linked to poorer adherence. Treatment fatigue over time may reduce vigilance in medication routines, underlining the importance of continuous counseling and psychological support (Janjua et al., 2021). International evidence supports pharmacist-led interventions in improving adherence, and recent initiatives in China to expand clinical pharmacy services provide opportunities to strengthen polypharmacy management (Lum et al., 2020; Martínez‐Mardones et al., 2023). In the Chinese healthcare context, further strengthening of hospital-based clinical pharmacy services, integration of pharmacists into multidisciplinary teams, and development of national guidelines for polypharmacy management would be valuable steps to enhance medication safety and patient outcomes.

A closer look at item responses revealed widespread gaps in understanding the definition of polypharmacy, drug interactions, and handling of missed doses. Such deficits expose patients to potential adverse events and highlight systemic shortcomings in patient education (Koyama et al., 2020; Luokkamäki et al., 2021). Many participants also expressed concerns about dependency, cost, and prescription complexity, which may hinder adherence and require targeted interventions to align perceptions with evidence-based recommendations (Henssler et al., 2024; Keogh et al., 2022).

Overall, this study underscores the need for comprehensive strategies integrating patient education, family support, and improved provider–patient communication. Enhancing patients’ knowledge and addressing misconceptions, while ensuring consistent follow-up care, will be critical for promoting safer and more effective medication practices among those with cardiovascular comorbidities. This study has several limitations. First, the sample was entirely composed of hospitalized patients, which may have introduced selection bias. Inpatients generally have more severe conditions, greater comorbidity burden, and lower self-management ability compared with outpatients. Although inpatients were chosen to ensure higher data accuracy and completion—with researchers available to explain questionnaire items on site—this selection might limit the generalizability of the findings to broader outpatient populations. This characteristic has been clearly acknowledged as a study limitation, as inpatients usually have more complex treatment regimens, a higher risk of polypharmacy-related issues, and may not fully represent the general outpatient population. This sampling approach may also limit the generalizability of the findings to community-dwelling patients with chronic cardiovascular diseases. Second, as the study was conducted in a single hospital, the findings may not be generalizable to other healthcare settings. Third, patients’ health literacy, which strongly influences knowledge, attitudes, and practices related to medication use, was not assessed and may represent an important confounding factor. These issues should be acknowledged when interpreting the results.

## Conclusion

This study clearly demonstrates significant challenges in medication management among inpatients with chronic cardiovascular comorbidities, specifically revealing insufficient knowledge and negative attitudes concerning polypharmacy. These deficiencies directly correlate with notable gaps in patient adherence behaviors. Our findings underscore an urgent, unmet need for targeted, multidisciplinary educational interventions. Such programs must be strategically designed not only to enhance patients’ understanding of their complex drug regimens but also to positively shift their attitudes toward polypharmacy. Ultimately, improving these foundational cognitive and affective factors is critical for supporting safer, more consistent medication practices and optimizing long-term cardiovascular health outcomes.

## Data Availability

The original contributions presented in the study are included in the article/Supplementary Material, further inquiries can be directed to the corresponding author.

## References

[B1] Bahiru E. Hsiao R. Phillipson D. Watson K. E. (2021). Mechanisms and treatment of dyslipidemia in diabetes. Curr. Cardiol. Rep. 23, 26–6. 10.1007/s11886-021-01455-w 33655372

[B2] Boateng G. O. Neilands T. B. Frongillo E. A. Melgar-Quiñonez H. R. Young S. L. (2018). Best practices for developing and validating scales for health, social, and behavioral research: a primer. Front. Public Health 6, 149. 10.3389/fpubh.2018.00149 29942800 PMC6004510

[B3] Chae J. Cho H. J. Yoon S.-H. Kim D.-S. (2024). The association between continuous polypharmacy and hospitalization, emergency department visits, and death in older adults: a nationwide large cohort study. Front. Pharmacol. 15, 1382990. 10.3389/fphar.2024.1382990 39144630 PMC11322047

[B4] Chantzaras A. Yfantopoulos J. (2023). Association between medication adherence and health-related quality of life of patients with hypertension and dyslipidemia. Horm. (Athens) 22, 665–676. 10.1007/s42000-023-00471-5 37493942

[B5] Clark C. M. Hejna M. Shao E. Maerten-Rivera J. L. Monte S. V. Wahler R. G. Jr (2020). Knowledge and attitudes of student pharmacists regarding polypharmacy and deprescribing: a cross-sectional study. Pharm. (Basel) 8, 220. 10.3390/pharmacy8040220 33217927 PMC7711500

[B6] Cross A. J. Elliott R. A. Petrie K. Kuruvilla L. George J. (2020). Interventions for improving medication-taking ability and adherence in older adults prescribed multiple medications. Cochrane Database Syst. Rev. 5, Cd012419. 10.1002/14651858.CD012419.pub2 32383493 PMC7207012

[B7] Goretzko D. Siemund K. Sterner P. (2024). Evaluating model fit of measurement models in confirmatory factor analysis. Educ. Psychol. Meas. 84, 123–144. 10.1177/00131644231163813 38250508 PMC10795573

[B8] Halli-Tierney A. D. Scarbrough C. Carroll D. (2019). Polypharmacy: evaluating risks and deprescribing. Am. Fam. Physician 100, 32–38. 31259501

[B9] Henssler J. Schmidt Y. Schmidt U. Schwarzer G. Bschor T. Baethge C. (2024). Incidence of antidepressant discontinuation symptoms: a systematic review and meta-analysis. Lancet Psychiatry 11, 526–535. 10.1016/s2215-0366(24)00133-0 38851198

[B10] Hsu H. F. Chen K. M. Belcastro F. Chen Y. F. (2021). Polypharmacy and pattern of medication use in community‐dwelling older adults: a systematic review. J. Clin. Nurs. 30, 918–928. 10.1111/jocn.15595 33325067

[B11] Huang Y.-T. Steptoe A. Wei L. Zaninotto P. (2021). Polypharmacy difference between older people with and without diabetes: evidence from the English longitudinal study of ageing. Diabetes Res. Clin. Pract. 176, 108842. 10.1016/j.diabres.2021.108842 33933497

[B12] Hussain A. S. M. Ghadzi S. M. S. Sulaiman S. A. S. Alsahali S. M. Khan S. F. (2025). Medication reconciliation: impact of an educational intervention on the knowledge, attitude and practices of healthcare professionals-a prospective quasi-experimental study in a Saudi referral hospital. J. Health. Popul. Nutr. 44, 15. 10.1186/s41043-025-00751-3 39844331 PMC11755839

[B13] Janjua S. Pike K. C. Carr R. Coles A. Fortescue R. Batavia M. (2021). Interventions to improve adherence to pharmacological therapy for chronic obstructive pulmonary disease (COPD). Cochrane Database Syst. Rev. 9, Cd013381. 10.1002/14651858.CD013381.pub2 34496032 PMC8425588

[B14] Jarrah M. Khader Y. Alkouri O. Al-Bashaireh A. Alhalaiqa F. Al Marzouqi A. (2023). Medication adherence and its influencing factors among patients with heart failure: a cross sectional study. Med. Kaunas. 59, 960. 10.3390/medicina59050960 37241192 PMC10224223

[B15] Jokanovic N. Tan E. C. Dooley M. J. Kirkpatrick C. M. Bell J. S. (2015). Prevalence and factors associated with polypharmacy in long-term care facilities: a systematic review. J. Am. Med. Dir. Assoc. 16, 535.e1–12. 10.1016/j.jamda.2015.03.003 25869992

[B16] Kamau M. Nyanja N. Lusambili A. M. Shabani J. Mohamoud G. (2024). Knowledge, attitudes and beliefs toward polypharmacy among older people attending family medicine clinic, Nairobi, Kenya. BMC Geriatr. 24, 132. 10.1186/s12877-024-04697-9 38317102 PMC10845745

[B17] Keogh B. Murphy E. Doyle L. Sheaf G. Watts M. Higgins A. (2022). Mental health service users experiences of medication discontinuation: a systematic review of qualitative studies. J. Ment. Health 31, 227–238. 10.1080/09638237.2021.1922644 34126035

[B18] Koyama A. K. Maddox C. S. Li L. Bucknall T. Westbrook J. I. (2020). Effectiveness of double checking to reduce medication administration errors: a systematic review. BMJ Qual. Saf. 29, 595–603. 10.1136/bmjqs-2019-009552 31391315 PMC7362775

[B19] Liu S. Li Y. Zeng X. Wang H. Yin P. Wang L. (2019). Burden of cardiovascular diseases in china, 1990-2016: findings from the 2016 global burden of disease study. JAMA Cardiol. 4 (4), 342–352. 10.1001/jamacardio.2019.0295 30865215 PMC6484795

[B20] Lum M. V. Cheung M. Y. Harris D. R. Sakakibara B. M. (2020). A scoping review of polypharmacy interventions in patients with stroke, heart disease and diabetes. Int. J. Clin. Pharm. 42, 378–392. 10.1007/s11096-020-01028-x 32319017

[B21] Luokkamäki S. Härkänen M. Saano S. Vehviläinen-Julkunen K. (2021). Registered nurses' medication administration skills: a systematic review. Scand. J. Caring Sci. 35, 37–54. 10.1111/scs.12835 32168398

[B22] Martínez‐Mardones F. Benrimoj S. I. Ahumada‐Canale A. Plaza‐Plaza J. C. Garcia‐Cardenas V. (2023). BC clinical impact of medication reviews with follow‐up in cardiovascular older patients in primary care: a cluster‐randomized controlled trial. Br. J. Clin. Pharmacol. 89, 2131–2143. 10.1111/bcp.15682 36735853

[B23] Morgado F. F. R. Meireles J. F. F. Neves C. M. Amaral A. C. S. Ferreira M. E. C. (2017). Scale development: ten main limitations and recommendations to improve future research practices. Psicol. Reflexão Crítica 30, 3. 10.1186/s41155-016-0057-1 32025957 PMC6966966

[B24] Naing L. Winn T. Rusli B. (2006). Practical issues in calculating the sample size for prevalence studies. Archives Orofac. Sci. 1, 9–14.

[B25] Remelli F. Ceresini M. G. Trevisan C. Noale M. Volpato S. (2022). Prevalence and impact of polypharmacy in older patients with type 2 diabetes. Aging Clin. Exp. Res. 34, 1969–1983. 10.1007/s40520-022-02165-1 35723858 PMC9464133

[B26] Roth G. A. Mensah G. A. Johnson C. O. Addolorato G. Ammirati E. Baddour L. M. (2020). Global burden of cardiovascular diseases and risk factors, 1990–2019: update from the GBD 2019 study. J. Am. Coll. Cardiol. 76, 2982–3021. 10.1016/j.jacc.2020.11.010 33309175 PMC7755038

[B27] Salman M. Mustafa Z. U. Rao A. Z. Khan Q. U. Asif N. Hussain K. (2020). Serious inadequacies in high alert medication-related knowledge among Pakistani nurses: findings of a large, multicenter, cross-Sectional survey. Front. Pharmacol. 11, 1026. 10.3389/fphar.2020.01026 32765259 PMC7381221

[B28] Shen J. G. N. Zheng S. (2024). Chinese expert consensus on polypharmacy assessment and management in the elderly. Chin. J. Geriatr. 43 (3), 269–278.

[B29] Tomida J. Wasa C. Jacobsen R. Revell J. H. P. Fujii A. Iihara N. (2024). Associated factors and causes of chronic disease medication oversupply. Biol. Pharm. Bull. 47, 2032–2040. 10.1248/bpb.b24-00551 39647907

[B30] Vainauskienė V. Vaitkienė R. (2021). Enablers of patient knowledge empowerment for self-management of chronic disease: an integrative review. Int. J. Environ. Res. Public Health 18, 2247. 10.3390/ijerph18052247 33668329 PMC7956493

