# Supplementary material for: Knowledge, attitudes, and practices of inpatients with chronic cardiovascular comorbidities regarding polypharmacy: a cross-sectional study
Source: Front Pharmacol. 2025 Nov 21;16:1702721. doi: 10.3389/fphar.2025.1702721 (PMC12678080; doi:10.3389/fphar.2025.1702721)
Supplement: Supplementary file 1 [file Supplementaryfile1.docx]

**Table S1 Distribution of Responses to Knowledge, Attitude, and Practice Items on Polypharmacy**

| **Knowledge** | | | **N (%)** | | |
| --- | --- | --- | --- | --- | --- |
|  |  |  | **Correct** | **Incorrect** | **Unaware** |
| 1. Polypharmacy generally refers to the routine use of five or more medications simultaneously. | | | 12(1.7) | 105(14.87) | 589(83.43) |
| 1. The more types of medications taken, the higher the risk of adverse reactions. | | | 182(25.78) | 353(50) | 171(24.22) |
| 1. Do you know the side effects of each medication you take? | | | 13(1.84) | 388(54.96) | 305(43.2) |
| 1. Do you know that interactions can occur between different medications? | | | 3(0.42) | 107(15.16) | 596(84.42) |
| 1. Do you know how to handle situations such as missed doses, incorrect doses, or repeated doses? | | | 7(0.99) | 236(33.43) | 463(65.58) |
| 1. Do you know that excessive use of medications can lead to adverse reactions? | | | 27(3.82) | 241(34.14) | 438(62.04) |
| 1. Are you aware that medication adjustments or changes should be discussed with a doctor? | | | 131(18.56) | 373(52.83) | 202(28.61) |
| **Attitude** | **Strongly agree** | **Agree** | **Neutral** | **Disagree** | **Strongly disagree** |
| 1. I believe using multiple medications simultaneously can better treat my disease. P | 25(3.54) | 295(41.78) | 221(31.3) | 160(22.66) | 5(0.71) |
| 1. I am satisfied with the number of medications I am currently taking. P | 5(0.71) | 195(27.62) | 234(33.14) | 262(37.11) | 10(1.42) |
| 1. If I stop taking these medications, my condition will worsen. N | 25(3.54) | 279(39.52) | 286(40.51) | 115(16.29) | 1(0.14) |
| 1. My life cannot function without these medications. N | 45(6.37) | 380(53.82) | 179(25.35) | 99(14.02) | 3(0.42) |
| 1. I believe medications should be taken strictly according to the doctor’s prescription, without arbitrary changes. P | 78(11.05) | 419(59.35) | 162(22.95) | 47(6.66) | / |
| 1. I think reducing the number of medications will better manage my health. N | 94(13.31) | 249(35.27) | 144(20.4) | 211(29.89) | 8(1.13) |
| 1. I find the prescriptions provided by the doctor too complicated to understand and remember. N | 46(6.52) | 229(32.44) | 195(27.62) | 225(31.87) | 11(1.56) |
| 1. I worry that taking so many medications will harm my liver and kidney function. N | 137(19.41) | 317(44.9) | 154(21.81) | 97(13.74) | 1(0.14) |
| 1. I think taking so many medications creates a financial burden for me. N | 49(6.94) | 277(39.24) | 118(16.71) | 257(36.4) | 5(0.71) |
| 1. I am concerned that long-term use of so many medications may lead to dependency. N | 84(11.9) | 202(28.61) | 234(33.14) | 182(25.78) | 4(0.57) |
| **Practice** | **Always** | **Often** | **Sometimes** | **Rarely** | **Never** |
| 1. I strictly follow the timing and frequency of medication as instructed by my doctor. P | 134(18.98) | 313(44.33) | 126(17.85) | 113(16.01) | 20(2.83) |
| 1. I forget to take my medication. N | / | 80(11.33) | 144(20.4) | 298(42.21) | 184(26.06) |
| 1. I have experienced worsening symptoms or adverse reactions because of missed or delayed medication. N | 1(0.14) | 46(6.52) | 179(25.35) | 181(25.64) | 299(42.35) |
| 1. I have interrupted my medication treatment due to forgetting to purchase or renew prescriptions. N | 1(0.14) | 41(5.81) | 107(15.16) | 209(29.6) | 348(49.29) |
| 1. I read the medication instructions to understand side effects and precautions. P | 17(2.41) | 40(5.67) | 125(17.71) | 164(23.23) | 360(50.99) |

“N” means negative, “P” means positive.

**Table S2. SEM fit indicators**

| **Indicators** | **Reference** | **Results** |
| --- | --- | --- |
| **Individuals taking five or more medications daily** | | |
| RMSEA | <0.08 Good | 0.000 |
| SRMR | <0.08 Good | 0.000 |
| TLI | >0.8 Good | 1.000 |
| CFI | >0.8 Good | 1.000 |
| **Individuals taking less than five medications daily** | | |
| RMSEA | <0.08 Good | 0.000 |
| SRMR | <0.08 Good | 0.000 |
| TLI | >0.8 Good | 1.000 |
| CFI | >0.8 Good | 1.000 |
