# Supplementary material for: Knowledge, attitudes, and practices of inpatients with chronic cardiovascular comorbidities regarding polypharmacy: a cross-sectional study
Source: Front Pharmacol. 2025 Nov 21;16:1702721. doi: 10.3389/fphar.2025.1702721 (PMC12678080; doi:10.3389/fphar.2025.1702721)
Supplement: Supplementary file 2 [file Supplementaryfile2.docx]

| **Knowledge, Attitudes, and Practices of Patients with Chronic Disease Comorbidities Regarding Polypharmacy** | | | |  |
| --- | --- | --- | --- | --- |
| Dear Participant:  We are researchers from **Taizhou Central Hospital**, sincerely inviting you to participate in our study. This research aims to understand the **knowledge, attitudes, and practices of patients with chronic disease comorbidities regarding polypharmacy,** providing a scientific basis for developing effective intervention strategies. This effort could help more people in the future improve their health. Participation in this study is voluntary. The study has been reviewed and approved by the Ethics Review Committee. If you agree to participate, please refer to the following instructions:  1.Please complete the questionnaire. There are no right or wrong answers; you only need to respond based on your actual situation. If you encounter any issues during the process, feel free to ask us. Once completed, please submit it promptly.  2.This is a simple questionnaire survey and will not harm your physical or mental health. However, it includes some personal questions, such as your gender and age. Please rest assured that your information will be kept strictly confidential and will not be disclosed.  3.As a participant, you have the right to inquire about relevant information and the progress of this study at any time. If you decide to withdraw from the study, please notify us, and your data will not be included in the results.  Finally, we sincerely thank you for taking the time to support our research!  □ I have been informed and agree to the collected data being used for scientific research.  Signature of informed consent:  Participation date: Year Month Day | | | |  |
| **Part 1 Basic Information** | | | | |
| Your gender: | | □a. Male | □b. Female | |
| Your age: (fill in an integer) | | | | |
| Place of residence: | | □Rural □Urban □Suburban | | |
| Education level: | | ☐ Primary school or below ☐ Middle school ☐ High school/Technical school ☐ Associate degree/Bachelor's degree or above | | |
| Your occupation:  ☐ Government/Enterprise Administrator ☐ Professional (e.g., teacher, doctor, engineer, writer, etc.) ☐ General Staff ☐ Business/Service Industry Personnel ☐ Production, Transportation, Equipment Operators ☐ Agricultural, Forestry, Animal Husbandry, Fishery, and Water Conservancy Workers ☐ Military Personnel ☐ Unemployed or Homemaker | | | | |
| Average monthly per capita income of your household in the past year (including in-kind income and rental income, etc.): ___ yuan □<2000 □2000-5000 □5000-10000 □10000-20000 □>20000 | | | | |
| Marital status: | | □Single □Married □Divorced □Widowed | | |
| Which of the following diseases do you have? (Multiple choice, at least two) | | ☐ Hypertension ☐ Diabetes ☐ Hyperlipidemia ☐ Coronary heart disease | | |
| Do any of your family members have similar chronic diseases? | | □Yes □No | | |
| Are you currently taking five or more medications daily? | | □Yes □No | | |
| Years you have been taking medication for chronic diseases: | | ☐ ≤1 year ☐ 2–5 years ☐ 6–9 years ☐ ≥10 years | |  |
| What medications are you currently taking?  ☐ Antihypertensive drugs: ____ types ☐ Antidiabetic drugs: ____ types ☐ Lipid-lowering drugs: ____ types ☐ Antiplatelet drugs (e.g., aspirin): ____ types ☐ Antianginal drugs: ____ types ☐ Other drugs: ____ types | | | |  |
| In the past six months, have you experienced adverse reactions during medication use? ☐ Yes ☐ No ☐ Uncertain | | | |  |
| Your type of health insurance:  ☐ New Rural Cooperative Medical Scheme ☐ Employee Medical Insurance ☐ Urban Resident Medical Insurance ☐ Commercial Medical Insurance ☐ No Insurance | | | |  |
| Do you have an attending physician or family doctor? | | □Yes □No | |  |

| **Part 2 Knowledge of Polypharmacy in Patients with Chronic Disease Comorbidities** | | | | | | | |
| --- | --- | --- | --- | --- | --- | --- | --- |
| For the following statements, please indicate your level of understanding: | | | | | | | |
| 1. Polypharmacy generally refers to the routine use of five or more medications simultaneously. | | a. very familiar | | b. partially familiar | | c. unaware | |
| 1. The more types of medications taken, the higher the risk of adverse reactions. | | a. very familiar | | b. partially familiar | | c. unaware | |
| 1. Do you know the side effects of each medication you take? | | a. very familiar | | b. partially familiar | | c. unaware | |
| 1. Do you know that interactions can occur between different medications? | | a. very familiar | | b. partially familiar | | c. unaware | |
| 1. Do you know how to handle situations such as missed doses, incorrect doses, or repeated doses? | | a. very familiar | | b. partially familiar | | c. unaware | |
| 1. Do you know that excessive use of medications can lead to adverse reactions? | | a. very familiar | | b. partially familiar | | c. unaware | |
| 1. Are you aware that medication adjustments or changes should be discussed with a doctor? | | a. very familiar | | b. partially familiar | | c. unaware | |
| **Part 3 Attitudes of Patients with Chronic Disease Comorbidities Towards Polypharmacy** | | | | | | | |
| 1. I believe using multiple medications simultaneously can better treat my disease. P | a. strongly agree | b. agree | c. neutral | | d. disagree | | e. strongly disagree |
| 1. I am satisfied with the number of medications I am currently taking. P | a. strongly agree | b. agree | c. neutral | | d. disagree | | e. strongly disagree |
| 1. If I stop taking these medications, my condition will worsen. N | a. strongly agree | b. agree | c. neutral | | d. disagree | | e. strongly disagree |
| 1. My life cannot function without these medications. N | a. strongly agree | b. agree | c. neutral | | d. disagree | | e. strongly disagree |
| 1. I believe medications should be taken strictly according to the doctor’s prescription, without arbitrary changes. P | a. strongly agree | b. agree | c. neutral | | d. disagree | | e. strongly disagree |
| 1. I think reducing the number of medications will better manage my health. N | a. strongly agree | b. agree | c. neutral | | d. disagree | | e. strongly disagree |
| 1. I find the prescriptions provided by the doctor too complicated to understand and remember. N | a. strongly agree | b. agree | c. neutral | | d. disagree | | e. strongly disagree |
| 1. I worry that taking so many medications will harm my liver and kidney function. N | a. strongly agree | b. agree | c. neutral | | d. disagree | | e. strongly disagree |
| 1. I think taking so many medications creates a financial burden for me. N | a. strongly agree | b. agree | c. neutral | | d. disagree | | e. strongly disagree |
| 1. I am concerned that long-term use of so many medications may lead to dependency. N | a. strongly agree | b. agree | c. neutral | | d. disagree | | e. strongly disagree |

| **Part 4 Practices of Patients with Chronic Disease Comorbidities Regarding Polypharmacy**  Always: Can do almost 100% of the time; Often: Can do more than 70% of the time; Sometimes: Can do 40–70% of the time  Rarely: Can do 10–40% of the time; Never: Can do less than 10% of the time | | | | | |
| --- | --- | --- | --- | --- | --- |
| 1. I strictly follow the timing and frequency of medication as instructed by my doctor. P | a. always | b. often | c. sometimes | d. rarely | e. never |
| 1. I forget to take my medication. N | a. always | b. often | c. sometimes | d. rarely | e. never |
| 1. I have experienced worsening symptoms or adverse reactions because of missed or delayed medication. N | a. always | b. often | c. sometimes | d. rarely | e. never |
| 1. I have interrupted my medication treatment due to forgetting to purchase or renew prescriptions. N | a. always | b. often | c. sometimes | d. rarely | e. never |
| 1. I read the medication instructions to understand side effects and precautions. P | a. always | b. often | c. sometimes | d. rarely | e. never |

| Thank you again for filling out our questionnaire, the information you provided will be valuable to us in the future!  Thank you for filling out our questionnaire！  If you have any comments and suggestions on this survey, we would be honored to hear your voice.  Opinions and Suggestions: (optional)  In order to this questionnaire research can actually play a role in promoting the smooth development of the future return visit, if you are willing to leave your contact information, we would be grateful!  Your phone number: (optional) |
| --- |
